# The Role of Psychological First Aid to Support Public Mental Health in the COVID-19 Pandemic

**DOI:** 10.7759/cureus.8821

**Published:** 2020-06-25

**Authors:** Kaushal Shah, Sukhmeet Bedi, Henry Onyeaka, Romil Singh, Gaurav Chaudhari

**Affiliations:** 1 Psychiatry, Griffin Memorial Hospital, Norman, USA; 2 Psychiatry, Harvard School of Public Health, Boston, USA; 3 Internal Medicine, Metropolitan Hospital, Jaipur, IND; 4 Psychiatry, Johns Hopkins Bloomberg School of Public Health, Baltimore, USA

**Keywords:** psychological first aid, coronavirus, mental health, pfa, pfa training, pfa models, covid-19, telepsychiatry, pfa efficacy, rapid

## Abstract

Psychological first aid (PFA) is one of the vital tools in delivering psychological interventions to those who have undergone or experienced traumatic events. Traumatic experiences during calamities, outbreaks of infections, and war can induce a significant amount of stress in the absence of early and effective intervention provided by trained caregivers. The coronavirus pandemic has caused significant levels of fear as governments impose quarantine and lockdown to contain the infection. Countries around the globe have halted several social and economic operations to curb the spread of coronavirus disease-19 (COVID-19). However, panic, helplessness, and horror aided by the infection due to the lack of a definitive cure has exposed the population to significant mental distress, thus warranting psychological intervention.

## Introduction and background

PFA is critical in ensuring the psychological recovery of people affected by disasters. PFA is a group of skills applied to limit distress and maladaptive behaviors that could negatively impact the rehabilitation of people from a traumatic experience [1]. Since its first conceptual introduction in the mid-twentieth century, PFA has been vital in availing early psychological intervention tools to survivors of natural calamities and devastating economic crises [2]. Key players such as American Red Cross (ARC) have identified various models and frameworks of PFA, such as coping in today's world, debriefs, stress first aid, and community-based psychological as essential in facilitating prompt psychological intervention. During the 2014 Ebola virus disease (EVD) outbreak in Liberia and Sierra Leone, PFA was pivotal in mounting psychological responses to the epidemic. The training-of-trainer (ToT) model, as well as the PFA guide provided by World Health Organization, enabled the education of healthcare workers, community leaders, teachers, and social workers in responding to the outbreak [3]. PFA's significance in mounting psychological intervention during a crisis makes it a beneficial tool for enhancing the mental well-being of trauma-stricken populations. 

The EMBASE, Google Scholar, and PubMed database were used to conduct the literature review by searching keywords "psychological first aid," "PFA," "psychological support," "counseling," in context of "mental health," "psychological distress," "emotional distress," "behavioral problem," "epidemic," "pandemic," "COVID-19", "coronavirus," "2019-nCoV", "infectious diseases," "Ebola," "SARS," "MERS," and "influenza." Articles found via this indexed search screened further to identify pertinent manuscripts or studies for our review. This manuscript provides in-depth coverage of various facets of PFA, including its significance, models, implementation and administration in epidemics, and training of health and non-health workers.

## Review

Overview

Psychological first aid is undoubtedly essential in enhancing the mental and emotional recovery of patients from traumatic experiences like disasters and pandemics. Disaster management organizations like the ARC are at the forefront of adopting PFA to help disaster survivors cope with the distress originating from traumatic experiences [3]. Psychological interventions are necessary for promoting resiliency and recovery of disaster survivors by reducing mental breakdown. Specifically, PFA enhances the coping of patients through various means, including dissemination of the right information, comforting, emotionally supporting, and fundamentally supporting disaster survivors, thus limiting mental health conditions like post-traumatic stress disorder (PTSD) [4].

Previously, emotional support was mostly provided by faith communities like churches to enhance the emotional and mental well-being of the population. However, since the 20th century, especially during World War II, there has been recognition of the necessity of psychological care through experts, as during the war it helped survivors in the recovery by alleviating distress through providing social support, meeting basic needs, and fostering immediate and long-term coping and functioning capacity [3]. Survivors of traumatic experiences need mental care to alleviate the impact of stressful events and accelerate recovery. Consequently, PFA's need to provide immediate and necessary psychological care is vastly significant in enhancing the resiliency of the population from trauma [3-5].

Mental health issues due to past infectious outbreaks 

Epidemics and outbreaks in the past have been known to induce various mental health issues due to trauma. The severe acute respiratory syndrome (SARS) outbreak ignited significant distress for healthcare workers and the general population [5,6]. Nearly a third to half of healthcare workers were distressed by the outbreak. Stigmatization, assigning doctors to new environments, and reducing interactions intensified social isolation hence facilitating trauma [6]. The risk of infecting the loved ones was also significant in increasing distress among healthcare workers. Depression, anxiety, drinking, smoking, and substance abuse intensified, while some healthcare workers started to miss their shifts due to the trauma stemming from the SARS outbreak. Quarantined staff was exhausted, detached, anxious, and indecisive after SARS [7]. The population under quarantine due to proximity and interacting with infected persons were distressed emotionally disturbed and distressed following the outbreak [7]. Families were fearful of losing their loved ones to the infection, while people experiencing similar symptoms associated with the disease, especially those with pregnant women and young children had higher emotional anxiety and disturbance [6,7].

Potential psychological issues due to coronavirus 

Coronavirus disease-19 (COVID-19) is one of the most significant threats currently facing many economies in various parts of the world. The massive case number of infection and deaths continues to rise despite the several stringent measures taken by governments to restrict its spread. Limiting measures such as isolation have negatively impacted the world population at a psychosocial level [4]. The prolonged incubation period of infection, along with its severity in older age groups, has promoted anxiety and fear amongst several families and individuals globally [5]. Several governments have imposed quarantine, while many others have imposed lockdown to contain the virus that is spreading at an alarming rate. Markets and economic activities in both developed and developing worlds have halted most of their operations to eradicate the infection [4,5].

The frontline healthcare personnel treating infected people are also negatively impacted by the infection because of the significant numbers of people needing treatment, thus inducing fatigue and anxiety. These current impacts can potentially cultivate mental health issues, especially on health workers. Multiple patients dying while in the care of skilled doctors, coupled with the horror and feeling of helplessness, can evolve into PTSD. The risk of infection, particularly on nurses responsible for handling infected people, can induce significant levels of stress and anxiety for them and their family members. The necessity of maintaining social distance as a preventive measure, limiting the interaction within and outside healthcare centers is further intensifying the psychological pressure. Consequently, the outbreak of COVID-19 will impact the mental health of a massive population of healthcare professionals involved in caring for the patients [4].

The challenges posed by COVID-19 have induced panic due to the lack of a known remedy for this virus. Families are now living in fear of being infected by the deadly virus as governments continue to fight it. Quarantine and lockdown measures are traumatizing people due to abrupt modifications in daily operations like work and learning [8,9].

Understanding the safety and efficacy of the PFA

PFA's application was effective during past outbreaks such as SARS, where the population was fearful and anxious about the severe infection. According to Gillespie (1963), PFA is a crucial psychological intervention tool for mitigating the adverse impacts of traumatic events of persons witnessing or surviving humanitarian disasters [8]. Physical, mental, behavioral, and spiritual reactions experienced by disaster survivors are potentially interfering with adaptive coping while impeding the recovery process [9]. The benefits of PFA extend to men and women in various cultures. One of PFA's basic standards is that it is culturally informed to enhance its effectiveness across many societies [10]. Safety and comfort are among the core actions of PFA considered vital to the population by the professionals in treating traumatic experiences. In the outbreak of SARS, PFA was influential in managing the stress and anxiety of both health workers and the population without causing adverse effects or deteriorating the existing condition of the people [6]. Similarly, the administration of PFA in students enhances their safety due to various distress symptoms that can prevail in school settings [11]. 

According to McCabe et al. (2011), individuals undertaking PFA training believe that the skills gained through the process are central in promoting disaster mental health principles and practices [12]. They explained that most participants agreed or strongly agreed on the necessity and outcomes of PFA. The perception concerning self-efficacy was equally high, owing to the benefits of applying psychologic intervention in incidences of trauma. PFA approach is promoted as a tool to help people help themselves, thus regaining control of their situation by identifying and using positive coping strategies [3]. The goal is motivating people to link internal and outside resources in meeting their needs to enhance their capacity to take action, which addresses their concerns. Consequently, PFA is effective in various groups within the population, including the medical staff, without any concerns regarding its adverse impact considering its safety aspect [3,12].

Core of several PFA models 

PFA models are significant in facilitating identification and response to patients undergoing traumatic experiences. According to Gillespie (1963), recognizing a person that is emotionally injured is the first and most fundamental principle in providing PFA [8]. John Hopkin's RAPID (Reflective Listening, Assessment, Prioritization, Intervention, and Disposition) PFA is one of the several models that address the needs of persons in distress due to exposure to trauma, disasters, and emergencies [1]. The acronym of the model, RAPID, provides core content of the training available to enhance the preparedness of the population to traumatic or emergencies that can incapacitate one's behavior during such experiences [12]. Reflective listening is the ability to utilize active listening techniques to establish and determine the vital aspects of the survivor's experience. Assessment entails determining the necessity of exploring the survivor's capacity in terms of mental or behavioral functioning through binary answers.

Further, it involves assessing the dimensional factors likely to facilitate or pose limitations to adaptive functioning and recoveries like understanding and following directions. Prioritization, a step after determining the patient's functional needs, is essential in guiding the intervention plan, especially the acute intervention plan where physical, psychological, and behavioral reactions are hindered. Also, the ability of survivors to perform daily necessary activities gets determined under prioritization [12]. Intervention is a vital step that comes after addressing the medical and physical needs of the patient. It helps in reducing acute distress by providing stress management techniques. This step is indispensable in facilitating the patient's emotional and behavioral recovery by assuring the patient, thus enabling the survivor to regain self-control by mitigating panic [8,9,12]. Disposition is the final procedure that involves determining whether the survivor restores functional capacities for engaging in daily basic and routine activities. A referral at this stage can be made to facilitate the recovery of the patient further. Consequently, John Hopkin's Model (RAPID) is influential in accommodating psychological intervention among trauma patients through step by step procedures from identifying survivors of a disaster to evaluating the recovery of the survivors and referring for additional treatment if necessary [12].

PFA training

Empowering the personnel of public health organizations by providing PFA training is vital in the disaster management preparedness to address the needs of persons in distress exposed to trauma, disasters, and emergencies [1]. The current challenges in times of emergency preparedness and management are contributed by the limited availability of skilled PFA professionals to offer services [9,10]. The training programs mounted by various models are beneficial in increasing qualified personnel and improving the skills of healthcare workers in availing the abilities needed to facilitate PFA during disasters and emergencies such as those witnessed in the outbreak of SARS. The anxiety and emotional distress occurring during outbreaks warrants adequate preparedness and training availed through PFA models [10].

Training in PFA is crucial in ensuring the availability of individuals capable of providing the necessary psychological intervention in the event of disasters such as fire, explosion, or disease outbreak. Moreover, PFA training ensures the population's preparedness from potential emergencies that can induce traumatic experiences and severe impacts absent timely intervention. Disaster can leave more people with psychological consequences compared to the number of people with various physical injuries. The potential millions of individuals left with psychological injuries necessitate numerous professionals with adequate training to intervene in promoting the resiliency of those affected by the emergency or disaster. The RAPID model, in particular, is rewarding by availing training to various people, thus equipping them with the essential skills and expertise to provide PFA during emergencies or traumatic situations. Through training, the population, including health workers, will be equipped with vital skills to intervene in mitigating trauma among disaster survivors [10,13].

Administering settings for the PFA

A person with basic training can provide PFA on disaster survivors. According to Brown et al. (2009), PFA, like medical first aid, does not necessitate the involvement of mental health clinicians with advanced medical training [9]. Healthcare workers like nurses are capable of providing the needed psychological intervention to their patients in attempts to facilitate resiliency and behavioral recovery [13]. It is, however, imperative to ensure the person providing a psychological response is prepared and trained in terms of the fundamental expertise in managing trauma among disaster patients. Individuals under mental stress can demonstrate a variety of reactions that demand adequate preparation of the person providing aid in terms of what to do or not. Normal reactions with the survivors exhibiting symptoms like trembling and muscular tension usually necessitate giving assurance and motivating the victim to enable them to regain composure [8]. However, some reactions require the involvement of highly trained and qualified personnel like psychiatrists, especially in extreme cases, and managing severe impacts of the trauma such as PTSD. These skilled professionals will ensure the rehabilitation of patients suffering from intense trauma in a gradual process involving multiple sessions with the survivors. Patients with panic reactions, for example, are less likely to respond to PFA due to problems with inattention, thus necessitating the intervention of a skilled advanced medical professional [8].

Given the time-sensitivity of psychological intervention, most PFA occurs at the site or near the trauma location. According to a study by Allen et al., PFA was most often provided in the closest sites to trauma site, such as shelters (78%), community centers (8%), hospitals (12%), survivors' homes (10%), or through telephone crisis lines (8%) [10]. Nearly 80% of the psychological trauma during the Korean conflict was restored in the combat zone [8]. Individuals with PFA training can cater for the disaster survivors where the disaster has occurred to ensure faster resiliency of the patient. Mild cases of trauma such as normal reaction which does not require extensive treatment can be administered faster at the site of the incident, thus saving time while enhancing the intervention's effectiveness. However, in some cases, psychological intervention occurs in health facilities. However, in cases where the survivor has medical and physical injuries, the intervention is only feasible in the hospital after addressing the physical and medical conditions. Consequently, the PFA can occur in various environments depending on the prevailing conditions and kind of reaction of the survivor [8,13].

Role of telepsychiatry 

The contribution of technologies extends the provision of psychological intervention in disaster survivors. The emergence of telemedicine or telepsychiatry is due to new technologies that can augment behavioral recovery in individuals undergoing traumatic experiences. In particular, Burnet Project has been vital in developing information society to facilitate medical and psychological intervention among patients with burn traumas [14]. The project is intensively involved in preventing and treating burns while facilitating functional and psychological recovery by interconnecting Burn Centers (BC) through an internet network in the Mediterranean area. It optimized the treatment of burn survivors before transferring to BC by coordinating interactions between BC and emergency treatment centers in various hospitals. Therefore, telemedicine or telepsychiatry is significant in facilitating rapid and effective psychological intervention in disaster survivors [13,14].

Results from the past implementation of PFA

The application of PFA during the past epidemics provided significant benefits in aiding people with emotional distress. Folkman and Greer's framework showed rewards in managing the psychological effects of the SARS outbreak. The extensive measure imposed at the time to curb the spread of the infection to other members of the population mounted mental stress across the community. It helped in coping with stressors by enabling a person to solve a particular problem and take control of the situation. Emotion-based coping enhances support and mitigates stress due to isolation. The emotional support provided to health workers was vital in reducing stress and alleviating mental anxiety stemming from interpersonal separation induced by the pandemic. Meaning-based coping was also essential in events whose resolution was yet realized by the healthcare workers [6]. Like Folkman and Greer's framework, PFA allowed psychological intervention and showcased benefits. In the outbreak of influenza, various people were trained as a means to prepare and protect the population [3,7]. The training program ensured the responders' availability to provide timely counseling and physiological intervention to both medical personnel and the community [15].

PFA in coronavirus outbreak

The role of PFA in the COVID-19 outbreak is crucial and significant in managing the intensifying mental stress originating from enormous impacts promoted by the infection in various facets of socioeconomic activities [4,5]. The growing mortality and morbidity across many economies instill fear, helplessness, and horror in both the infected and uninfected populations. In the past, international organizations like the World Health Organization (WHO) has shown benefits by implementing PFA to reduce panic and anxiety within pandemic stricken locations like Ebola in Liberia and Sierra Leone. Local and international organizations should play an imperative role in increasing government efforts to safeguard the health of people [8].

Training of additional staff in hospitals and trauma centers to deal with the panic induced by the pandemic is among the first step in realizing the benefits PFA. One of the current gaps challenging the effectiveness of PFA is the scarcity of skilled personnel with PFA skills to facilitate psychological intervention [8,16-22]. Given the rising infection rates and statistics concerning COVID-19, several people will need psychological intervention to help conform and cope in the new environment [4-5]. The intensifying levels of fear among the public due to the treatment dilemma require a large number of trained personnel with skill in psychological intervention. Approaches such as train-the-trainer (TTT) and just-in-time (JIT) models will be rewarding in mounting training exercises to increase the number of providers capable of rendering psychological intervention due to coronavirus [15].

Educating the current medical staff on managing stress through PFA will help them cope with the government's rapid measures, such as quarantine and limited physical contact in workplaces and homes. Self-isolation, a means of limiting the spread of the infection, proved to induce distress due to stigmatization [15,16]. Increasing the number of individuals with qualifications in administering psychological intervention in health workers, infected patients, and affected populations will extend PFA's reach and effectiveness in mitigating trauma stemming from the COVID-19 pandemic [19]. The need for psychological intervention will rise after containing or eradicating coronavirus, as most of the nurses, physicians, residents, fellows, and healthcare workers were ill-equipped to deal with the mental stress stemming from the outbreak [8]. With limited psychological intervention skills, healthcare workers may be unprepared to deal with the psychological toll during and after the pandemic. Imparting PFA skills through training programs will prepare people psychologically, thus reducing distress within the workforce and population as a whole [21-23].

International organizations will be at the forefront in mounting PFA implementation. Organizations like WHO have demonstrated success in incorporating PFA to facilitate psychological intervention during the past outbreaks. ARC already has PFA skills and experience, and this will be significant in deploying the much needed psychological intervention to combat mental issues originating from the coronavirus pandemic [3]. 

The PFA intervention should be extended to both private and public learning institutions to support people, especially the future young adults of tomorrow. Through partnership programs with schools and other learning institutions, local health departments and organizations hold significant value to ensure minimal panic, horror, and distress as governments continue to work on long-term solutions to the pandemic [19]. During the SARS outbreak, families with young ones were among the most psychologically impacted group in terms of anxiety and distress [8,20]. Creating awareness through schools concerning the efforts of the governments to protect the young population will assure families who may be concerned for their children's safety. Schools should also reassure their learners to calm them as a measure to incapacitate panic and helplessness originating from the pandemic [21,24]. 

The coronavirus pandemic is a global crisis, impacting socioeconomic activities in both developed and developing economies. Technology-based solutions, applications, and the internet can also increase digital interaction among the population at both national and international levels [21]. Telepsychiatry will allow psychiatrists, psychologists, and trained PFA personnel to reach more extensive coverage when delivering psychological interventions. Health workers at the forefront in fighting the infection will also receive family support without the risk of infecting their loved ones and vice versa, thus reducing their stress while enhancing positive coping mechanisms. Forming support groups through digital chatrooms and social media will enhance recovery and coping by keeping the population connected and socially active despite isolation measures like quarantine [21-26]. PFA administered through technology will ensure people's coping during the COVID-19 crisis and resiliency after resolving the pandemic. Negative impacts stemming from trauma, such as during the previous SARS outbreak, should be mitigated through the productive implementation of PFA in combating the current crisis [21-24,27].

Moreover, soliciting help from faith communities would be beneficial in implementing and providing psychological intervention. In the past, religious institutions played a pivotal role in enhancing the emotional well-being of their believers by providing emotional assurances to the congregation under challenging times. Most importantly, several people have specific beliefs depending on their religions; hence awareness and campaign programs by faith institutions are crucial in mounting psychological interventions. The reassurance facilitated by faith communities in availing emotional support and calm during difficulties coincides with the principles of PFA. PFA comforts people and helps them remain calm, thus promoting mental well-being [23-26]. By calming the population with the help of faith communities, people undergoing distress and anxiety due to social isolation will be psychologically empowered to fight the pandemic [25].

## Conclusions

In summary, PFA is critical in delivering psychological interventions to survivors of a disaster. The negative implications of disease outbreak like fear, helplessness, and stress can expedite significant mental distress if not addressed promptly. The time sensitivity in trauma cases necessitates decisive and efficient measures to enable the patient to cope and regain baseline functional behavior. The application of PFA in the COVID-19 pandemic will protect the well-being of both healthcare personnel and the general population while augmenting the current treatments and measures to manage the outbreak. PFA aids in managing the psychological distress emanating from the infection's frights due to deaths and the stagnation of socioeconomic activities globally. Consequently, the application of PFA through training people on psychological intervention, coupled with the implementation of technology to enhance socialization and support, will help people cope better during and after the pandemic.
